# Association of COVID-19 Incidence and Mortality Rates With School Reopening in Brazil During the COVID-19 Pandemic

**DOI:** 10.1001/jamahealthforum.2021.5032

**Published:** 2022-02-11

**Authors:** Guilherme Lichand, Carlos Alberto Doria, João Paulo Cossi Fernandes, Onicio Leal-Neto

**Affiliations:** 1 Department of Economics, University of Zurich, Zurich, Switzerland; 2 Inter-American Development Bank, Brasilia, Brazil

## Abstract

**Importance:**

School closures because of COVID-19 have left 1.6 billion students around the world without in-person classes for a prolonged period. To our knowledge, no study has documented whether reopening schools in low- and middle-income countries during the pandemic was associated with increased aggregate COVID-19 incidence and mortality with appropriate counterfactuals.

**Objective:**

To test whether reopening schools under appropriate protocols during the COVID-19 pandemic was associated with increased municipal-level COVID-19 cases and deaths in São Paulo State, Brazil.

**Design, Setting, and Participants:**

This observational study of municipalities in São Paulo State, Brazil, uses a difference-in-differences analysis to examine the association between municipal decisions to reopen schools during the COVID-19 pandemic and municipal-level COVID-19 case and death rates between October and December 2020. The study compared 129 municipalities that reopened schools in 2020 with 514 that did not and excluded data for 2 municipalities that reopened schools and closed then again.

**Main Outcomes and Measures:**

New COVID-19 cases and deaths per 10 000 inhabitants up to 12 weeks after school reopenings and municipal-level aggregate mobility for a subset of municipalities.

**Results:**

There were 8764 schools in the 129 municipalities that reopened schools compared with 9997 in the control group of 514 municipalities that did not reopen schools. The municipalities that reopened schools had a cumulative COVID-19 incidence of 20 cases per 1000 inhabitants and mortality of 0.5 deaths per 1000 inhabitants in September 2020 (the baseline period) compared with an incidence of 18 cases per 1000 inhabitants and mortality of 0.45 deaths per 1000 inhabitants during the baseline period in the comparison group. The findings indicated that there were no statistically significant differences between municipalities that authorized schools to reopen and those that did not for (1) weekly new cases (difference-in-differences, –0.03; 95% CI, –0.09 to 0.03) and (2) weekly new deaths (difference-in-differences, –0.003; 95% CI, –0.011 to 0.004) before and after October 2020. Reopening schools was not associated with higher disease activity, even in relatively vulnerable municipalities, nor aggregate mobility.

**Conclusions and Relevance:**

The findings from this study suggest that keeping schools open during the COVID-19 pandemic did not contribute to the aggregate disease activity.

## Key Points

**Question:**

Is the reopening of schools during the COVID-19 pandemic associated with increased COVID-19 incidence and mortality?

**Findings:**

In this cross-sectional study of 643 Brazilian municipalities including 18 761 schools, on average, there was no systematic association between school reopening and COVID-19 incidence or mortality in São Paulo State up to 12 weeks after reopening, which was also the case for schools in the most vulnerable conditions. Aggregate mobility was already high before the school reopening and did not significantly increase afterwards.

**Meaning:**

The results of this study suggest that reopening schools under appropriate protocols in low- and middle-income countries during the pandemic is unlikely to be associated with higher aggregate COVID-19 cases or deaths when counterfactual mobility is already high.

## Teaser

This cross-sectional study examines whether reopening schools under appropriate protocols during the COVID-19 pandemic was associated with increased municipal-level COVID-19 cases and deaths in São Paulo State, Brazil.

## Introduction

Most countries around the world closed schools to reduce COVID-19 infections for a prolonged period.^1,2^ Beyond learning outcomes, school closures have also been shown to have an adverse association with children’s well-being^1,2,3,4^ and to increase dropouts.^5,6^

In-person classes might contribute to COVID-19 incidence and mortality. First, recent evidence suggests that the likelihood of infection among family members and school staff increases substantially when schools are open.^7,8^ Second, nonpharmaceutical measures have been shown to contribute to slowing down disease activity.^9,10,11,12,13,14,15^ School closures were typically included in these interventions, as they are more densely populated than most other establishments.^16,17,18,19^ Third, the mobility of primary caregivers is expected to increase when children are at school, potentially increasing transmission and beyond the school setting.^20,21^ The risks of in-person classes might be especially high in low- and middle-income countries where schools may lack resources for robust mitigation strategies that reduce transmission.^22,23,24^

Conversely, keeping schools open during the pandemic might not elevate risks of COVID-19 incidence and mortality. In fact, studies suggest that children, particularly those in primary school, are among the safest groups for whom social distancing could be gradually forgone because of their relatively low transmission risk.^18^ To our knowledge, no study has documented the aggregate consequences of keeping schools open in low- and middle-income countries during the COVID-19 pandemic with appropriate counterfactuals. Few studies have examined the association between school reopenings during the pandemic and COVID-19 disease activity in high-income countries,^25,26,27^ and their findings were mixed.

This article examines a natural experiment in São Paulo State, Brazil. All state schools were closed by the end of March 2020 in response to increasing COVID-19 cases. In-person school activities remained suspended throughout September 2020. Between October and December 2020, 129 municipalities authorized schools to partially reopen for in-person activities. Using public data on the evolution of the pandemic across different municipalities, we tested whether school reopenings under appropriate protocols was associated with increased COVID-19 cases and deaths in São Paulo State, considering the staggered timing of municipal decrees that authorized schools to reopen in the state.

## Methods

### Data Sources

The study was deemed exempt from institutional review board approval because it only used data on publicly available health outcomes that were aggregated at the municipality level. As such, no individual data were analyzed. First, we used data on the timing of municipal decrees that authorized schools to partially reopen for in-person activities in the state that were collected by the São Paulo State Secretariat of Education. Second, we used publicly available data on COVID-19 cases and deaths from municipal health secretariats and the Brazil.io repository.^28^ We focused on the period ranging from August to December 2020 so that we could examine trends for COVID-19 cases and deaths before and (up to 12 weeks) after school reopenings in the state. Third, we collected data for aggregate mobility from Google reports, which were available only for a subset of all municipalities. Fourth, we collected municipality characteristics (eg, per capita income and population) from Brazilian Census data. Lastly, we collected school characteristics from the Brazilian school census. We provide more details about data sources in eTables 1 and 2 of the Supplement and variable descriptions in the eMethods in the Supplement.

### Outcomes

We focused on 2 main outcomes of interest: new weekly per capita COVID-19 cases and deaths (both in logs). The log transformation allowed us to interpret the estimates as percentage differences in the outcomes of interest. We added 1 to all values to prevent dropping weekly observations for which we did not observe any new cases or deaths. As such, the study contained values for both outcomes across all municipalities and periods in the sample.

### Exposure

This study contains data on the existence and timing of all municipal decrees that allowed schools to partially reopen in São Paulo State since October 2020. A total of 131 of the 645 municipalities (20.3%) in the state eventually reopened schools in 2020 (eFigure 1 in the Supplement). Of those, we excluded 2 municipalities from the analysis that authorized schools to reopen at first but reversed that decision shortly thereafter. Schools that reopened had to follow safety protocols; in particular, all school staff had to wear personal protective equipment, hand sanitizer had to be made available at the school gate, and in-person attendance was limited (eg, at 35% capacity in regions where the severity of the pandemic was high).

In this study, we compared the trends of the outcomes of interest within municipalities that authorized schools to reopen with those that did not before and after authorization decrees. We do not have data on how many students actually attended in-person classes during that period; as such, we were only able to estimate intention-to-treat effects of school reopening. The São Paulo Secretariat of Education estimated that approximately 2 million students (of the 3.5 million public school students in the state) attended in-person school activities between October and December 2020.^29^ A companion study estimated that school reopenings significantly increased student attendance in the State.^6^

### Statistical Analyses

Because decisions whether to reopen schools for in-person activities in 2020 were not random, merely comparing trends of COVID-19 cases and deaths across different municipalities after in-person activities were allowed to resume would mistakenly associate school reopenings with disease activity, especially when considering mean reversion.

 To deal with this challenge, we implemented a difference-in-differences strategy that parsed out any baseline differences across municipalities as long as those that eventually authorized schools to reopen and those that would not have exhibited systematic differences in trends for the outcomes of interest in the absence of school reopenings. For each cohort of municipalities that reopened schools (defined by the week at which their authorization decree was made effective), we formally estimated the association between school reopenings and disease activity using the difference-in-differences estimator of Callaway and Sant’Anna.^30^ Because this estimator includes a matching procedure in the first step (see the eAppendix in the Supplement), the parallel trends assumption has to hold only conditionally for its difference-in-differences estimates to be valid. Thus, in the absence of school reopening, the growth rates of COVID-19 cases and deaths should have been the same across municipalities that did or did not authorize schools to reopen in 2020. While this is an assumption of the method, we could assess it implicitly by testing whether, conditional on baseline characteristics, the outcomes of interest exhibited differential trends across the 2 groups before October 2020 (when no municipalities had yet authorized in-person activities to resume) (eFigure 2 in the Supplement). Because in-person activities returned at different weeks across cohorts, we first estimated cohort-specific difference-in-differences coefficients through ordinary least squares and then combined them by weighting each estimate by its relative group size when computing the aggregate association for the whole sample.^30^

For all regressions, we controlled for municipal characteristics (income, population, number of schools, number of students per 1000 inhabitants, and the average quality of school infrastructure) from the 2010 Brazilian population census and the baseline per capita COVID-19 cases and deaths (cumulative between January and September 2020, immediately before school reopening). School infrastructure was the first principal component from a vector of school characteristics from the 2019 Brazilian school census (whether the school had a working kitchen, working bathrooms, trash collection, adequate sanitation infrastructure and access to drinking water, and its average class size).

We also estimated heterogeneous associations between school reopenings and COVID-19 disease activity regarding the following municipality characteristics: (1) baseline COVID-19 deaths; (2) average quality of school infrastructure; (3) per capita income; and (4) share of the population 65 years or older. In each case, we split municipalities according to the sample median and focused on whether the associations were statistically different from 0 within the highest risk/most vulnerable subsample.

Lastly, we estimated the association between school reopenings and the municipality’s average mobility index during that week using the same difference-in-differences strategy. This outcome is publicly available from Google mobility reports^31^ based on cell phone location. The outcome we had access to is an index that captures changes in mobility compared with the municipal average during the week of February 15, 2020, as measured in percentage points. The index is only available for 412 municipalities in São Paulo State. While these mobility data were not tagged to the school-relevant population, the question was whether authorizing schools to reopen had an association with overall mobility in each municipality greater and beyond that of children and their caregivers in tandem with the main analyses, which concerned municipal-level COVID-19 cases and deaths. Analyses were conducted using Stata (StataCorp) and R (R Foundation).

## Results

This study included 643 municipalities in São Paulo State, of which 129 (20.1%) authorized schools to reopen in 2020 (comprising 8764 schools) and 514 (79.9%; 9997 schools) did not. Municipalities that reopened schools had a cumulative COVID-19 incidence of 20 cases per 1000 inhabitants and mortality of 0.5 deaths per 1000 inhabitants during the baseline period. Municipalities that did not authorize schools to reopen had a cumulative COVID-19 incidence of 18 cases per 1000 inhabitants and mortality of 0.45 deaths per 1000 inhabitants during the same period. eTable 8 in the Supplement presents descriptive statistics for municipalities that authorized schools to reopen in 2020 and those that did not. On average, municipalities that reopened schools were larger, richer, and had fewer new COVID-19 cases and deaths before October 2020 than those that did not.

**Figure 1.**
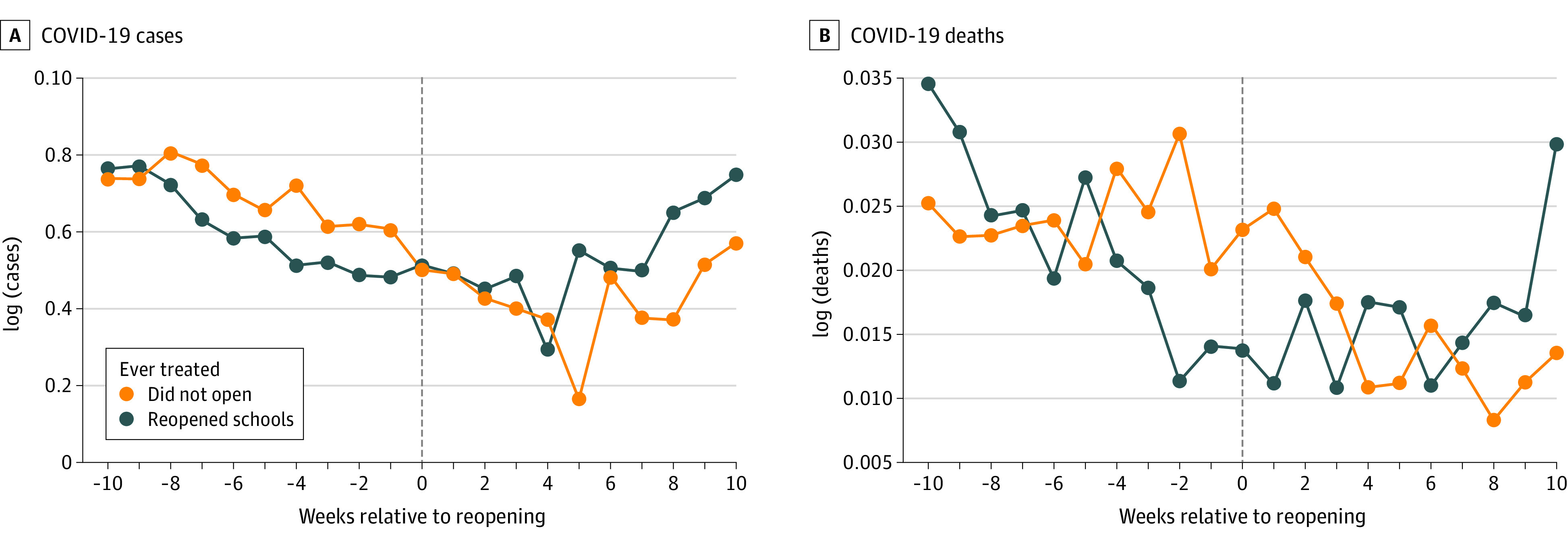
Trends in the Outcomes of Interest for Municipalities That Reopened Schools and Those That Did Not Trends for the outcomes of interest separately for municipalities that reopened schools and those that did not. Log of cases (A) and log of deaths (B) per 10 000 inhabitants. For the control group, the week of reopening was normalized to the last week of September 2020. The sample included 129 municipalities for the schools that reopened and 514 for the control group (schools that did not reopen).

**Figure 2.**
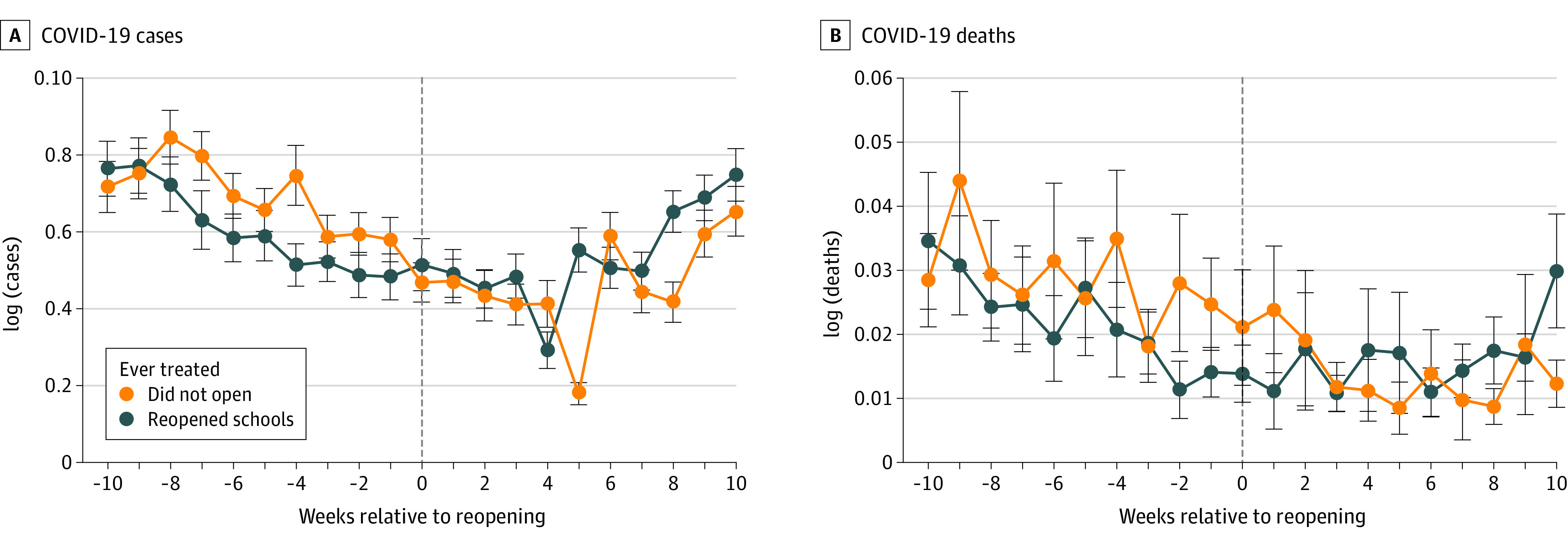
Trends in the Outcomes of Interest for Municipalities That Reopened Schools and Those That Did Not With a Matched Sample Trends for the outcomes of interest separately for municipalities that reopened schools and those that did not and were the closest matches for the municipalities that reopened. Log of cases (A) and log of deaths (B). For the control group, the week of reopening was normalized to the last week of September 2020; the vertical bars represent 95% CIs. The sample included 129 municipalities for each group, those with schools that reopened and the control group (those with schools that did not).

Figure 1 presents trends in the log of COVID-19 cases and deaths per 10 000 inhabitants separately for each group of municipalities. We observed no clear acceleration trend in either new cases or deaths for the municipalities that reopened schools. Figure 2 presents trends for those outcomes for the subsample that most closely matched the characteristics of municipalities that reopened schools in the control group. For this matched sample, trends were even more similar across groups.

**Figure 3.**
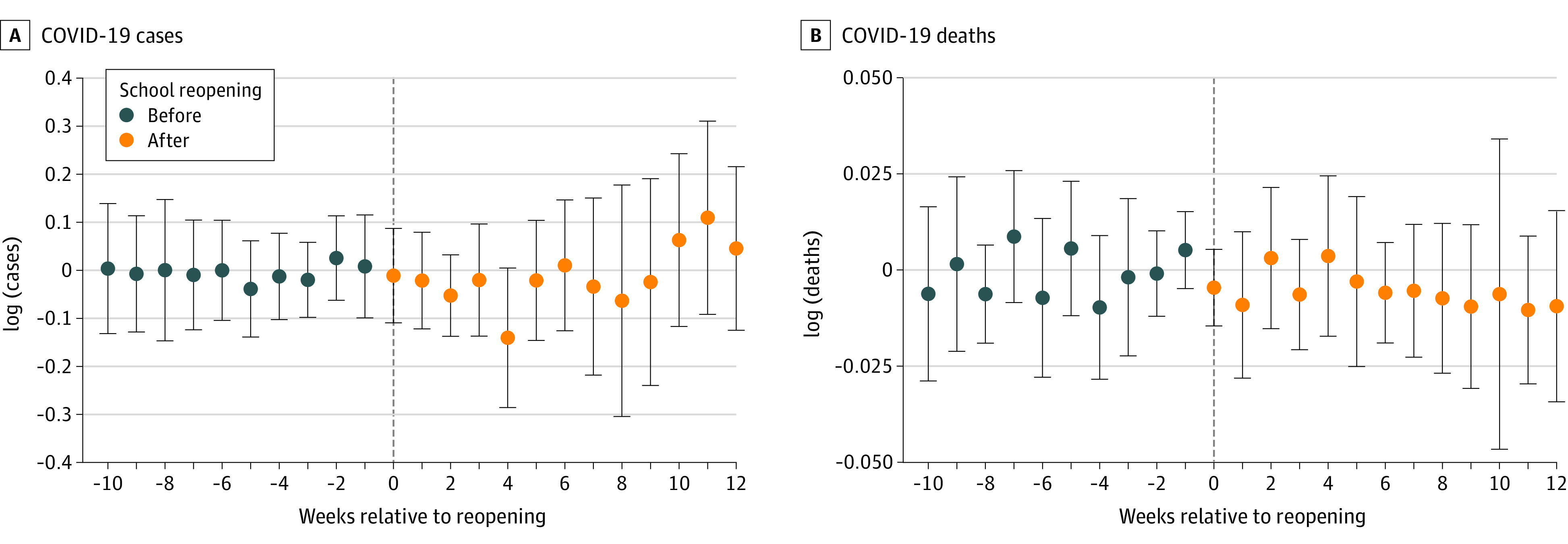
Estimates of the Difference Between the Groups Using the Difference-in-Differences Estimator Estimated difference between municipalities that reopened schools and those that did not using the Callaway and Sant’Anna estimator. Vertical bars represent 95% CIs.

Figure 3 presents estimates of the effects of school reopening nonparametrically, indicating difference-in-differences coefficients by week after school reopening for the log of new COVID-19 cases per 10 000 inhabitants and new COVID-19 deaths per 10 000 inhabitants. We estimated no statistically significant difference between the 2 groups either before (falsification tests) or after school reopening up to 12 weeks after in-person activities were allowed to resume.

In the Table, we compiled cohort-specific estimates of school reopenings on the log of new cases per 10 000 inhabitants, the log of new deaths per 10 000 inhabitants, and the aggregate mobility index, as estimated through difference-in-differences. We found no association between school reopenings and COVID-19 cases up to 12 weeks after reopening (–0.03; 95% CI, –0.09 to 0.03) for the log of weekly cases. Similarly, we found no association between school reopenings and COVID-19 deaths (–0.003; 95% CI, –0.011 to 0.004) for the log of weekly deaths. The average association with cases and deaths was negative. The data in the Table also suggest the value of the estimation technique, as there is substantial variation in cohort-specific estimates. Lastly, school reopenings were not associated with an increase in local mobility (1.465; 95% CI, –0.062 to 2.993), a small association that was not statistically different from 0 at conventional significance levels.

**Table.** Difference-in-Differences Estimates Aggregated by Cohorts^a^

Week of authorization decree	Log (95% CI)		Mobility
	Weekly COVID-19 cases	Weekly COVID-19 deaths	
October 5-9	0.002 (–0.071 to 0.076)	–0.009 (–0.019 to 0.001)	1.855 (–0.063 to 3.773)
November 2-6	–0.084 (–0.273 to 0.104)	0.015 (–0.024 to 0.054)	–0.023 (–3.989 to 3.943)
November 9-13	–0.080 (–0.207 to 0.046)	0.002 (–0.005 to 0.010)	0.112 (–2.878 to 3.102)
November 23-27	–0.121 (–0.290 to 0.048)	0.001 (–0.014 to 0.013)	4.761^b^ (1.985 to 7.536)
November 30-December 4	0.046 (–0.195 to 0.288)	–0.008 (–0.022 to 0.006)	0.006 (–5.061 to 5.072)
Aggregate difference	–0.030 (–0.086 to 0.026)	–0.003 (–0.011 to 0.004)	1.465 (–0.062 to 2.993)

^a^ This table shows the estimated difference between municipalities that reopened schools and those that did not using the Callaway and Sant’Anna estimator aggregated at the cohort level. It indicates point coefficients and 95% CIs.

^b^ *P* = .001.

The results in eTable 3 in the Supplement suggest no significant association between school reopenings and disease activity within municipalities most at risk, which were those with a less than median quality school infrastructure, less than median per capita income, greater than median senior population share (65 years or older), and greater than median baseline disease activity. Also, using additional COVID-19 testing data, which allowed for studying whether the association between school reopening and COVID-19 cases varied significantly by age, the data in eTable 4 in the Supplement indicate no statistical difference in cases between school-aged children and young adults across municipalities that authorized schools to reopen and those that did not before and after October 2020. The data in eTable 5 in the Supplement indicate no difference between individuals aged 28 to 48 years (within the typical age range of primary and secondary school parents) and 49 to 65 years using the same strategy. The study results were robust to matching municipalities across groups based on characteristics (eTable 6 in the Supplement) and estimating spillover effects from reopening decrees whereby changes in local new COVID-19 cases and deaths could be associated with school reopening decisions of neighboring municipalities (eTable 7 in the Supplement). eFigure 2 in the Supplement suggests that the study conclusions would remain unchanged if we used alternative measures of the outcome variables, such as excluding municipalities with 0 COVID-19 cases or deaths or using these variables in levels instead of logs.

**Figure 4.**
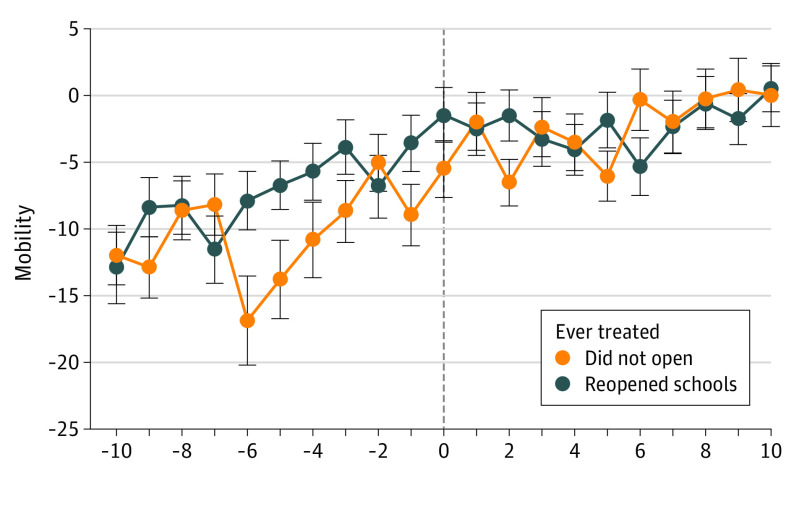
Trends in Mobility Index for the Matched Sample Trends for the Google mobility index separately for municipalities that reopened schools and those that did not and had similar characteristics as the former group. Details of the matching procedure are given in the eAppendix in the Supplement. The dependent variable represents weekly mobility compared with February 2020.

Figure 4 presents Google mobility data that show the index separately for each group of municipalities. For both groups, mobility was approximately 13 percentage points lower than prepandemic levels 12 weeks before school reopenings. Mobility evolved similarly across all municipalities, increasing sharply even before schools were allowed to reopen, and ultimately reaching prepandemic levels by late November even within municipalities that did not reopen schools in 2020.

## Discussion

The results of this cross-sectional study of 643 municipalities in São Paulo State suggest that reopening schools during the pandemic was not systematically associated with higher COVID-19 cases or deaths. The average differences in disease activity after October 2020 across municipalities that authorized schools to reopen and those that did not were small and not statistically significant at any week after reopening regarding new COVID-19 cases or deaths. This is not because the study design lacked statistical precision; effect sizes on cases were nearly 0 during most weeks of the study period, and the average COVID-19 incidence and mortality was higher within municipalities that did not reopen schools in 2020. The results suggest that school reopening was not significantly associated with disease activity even in municipalities with lower-quality school infrastructure, lower per capita income, or a higher senior population share or in those most severely affected by the pandemic. Evidence from the studies conducted in high-income countries about the association of school reopenings and COVID-19 disease activity is mixed,^25,26,27^ and the empirical strategies used could only estimate the short-term associations of school reopenings with disease activity (2-3 weeks after in-person classes returned).

In low- and middle-income countries, to our knowledge, most studies only analyzed risks within the school community rather than at the aggregate level,^32^ and none of them estimated effects under appropriate counterfactuals. Studies that estimate the effects of school reopening by comparing cases within municipalities before and after in-person activities returned are likely to detect false positives. This is because of mean reversion, the statistical tendency for negative (positive) shocks to be followed by positive (negative) ones.^31^ Locations that allow schools to reopen are statistically more likely to have experienced unusually low COVID-19 cases before in-person classes returned. Analogously, comparing the average COVID-19 incidence and mortality across locations that authorized schools to reopen with those that did not would potentially overestimate the effects of reopening during the pandemic; such locations typically differ in many dimensions, particularly regarding their previous trends for COVID-19 cases, hospitalizations, and deaths. Analyzing a comparison group with similar pretrends for COVID-19 case rates and deaths is a critical element of a study design to assign a clearer interpretation to empirical findings.

Resuming in-person school activities might potentially affect the aggregate evolution of the COVID-19 pandemic through 2 mechanisms. First, students and school staff might get infected at school and subsequently infect their families. Second, school reopenings might increase the mobility of children and their caregivers, leading to infections beyond the school setting. Regarding the first mechanism, schools were authorized to reopen in São Paulo State during the study period only under appropriate protocols, including strict limits to in-person attendance. For the second mechanism, the analysis of mobility data suggests that school reopening was not significantly associated with higher aggregate mobility in a setting where counterfactual mobility was already very high. The mobility index in São Paulo State reached prepandemic levels at the beginning of November, which suggests that families were not safe from contagion in the absence of in-person school activities.

### Limitations

There are 3 limitations to the analyses in this study. First, data limitations only allowed for documentation of the association between school reopenings and aggregate disease activity. As such, the null results estimated in this study do not imply that school reopenings during the COVID-19 pandemic pose no risks for school staff or the families of students, especially in settings where robust protocols to prevent infections at the school setting are not in place. Additional research is needed to document the direct effects of school reopenings in low- and middle-income countries on those populations with appropriate counterfactuals. That would require drawing on individual-level data on COVID-19 cases, hospitalizations, and deaths for students, school staff, and their families for schools that reopened and those that did not while parsing out any differences in previous trends in disease activity across them.

Second, we were only able to estimate intention-to-treat associations of school reopenings with disease activity. This is because we do not have detailed information on which schools effectively reopened or municipal-level school attendance. As such, this study’s findings are not informative about the association of marginal increases in student attendance with municipal-level COVID-19 cases and deaths.

Third, these findings might not necessarily replicate in other settings. Brazil is dense and was hit hard by the COVID-19 pandemic, which is likely a relevant benchmark for other low- and middle-income countries. Moreover, the null results documented in this study remain consistent across a wide range of local contexts, such as income levels, school infrastructure, senior population share, and local disease activity. However, in other countries or during periods in which mobility has been more successfully restrained by nonpharmaceutical measures, the study cannot rule out that school reopenings could be associated with increased overall mobility and contagion, contributing to aggregate disease activity, especially if safe reopening protocols are not in place. Complementary approaches, from seroprevalence surveys^33,34^ to identifying which variants of the virus are circulating in these settings,^35^ are also needed to further inform risk assessments in each context.

## Conclusions

The findings in this study suggest that reopening schools in low- and middle-income countries during the pandemic is unlikely to contribute to aggregate risk in the presence of safe reopening protocols, especially where mobility is already high. As such, the results suggest that the aggregate benefits of keeping schools closed are low. Together with recent evidence about the large educational costs of school closures across countries of all income levels,^6,36^ the results suggest that the policy debate in low- and middle-income countries must focus on how to safely keep schools open amidst the pandemic rather than whether or not to do so.

## Notes

Supplement. **eTable 1.** Summary of data sources **eTable 2.** Variable description
eMethods.

eAppendix.
**eFigure 1. **Timeline of school reopening **eTable 3. **Heterogeneous treatment effects estimated through difference-in-differences **eTable 4. **Differences in cases for school-age children aggregated by cohort **eTable 5. **Differences in cases for parent-age cadults aggregated by cohort **eTable 6. **Difference-in-difference estimates aggregated by cohorts with matched sample **eTable 7. **Difference-in-difference model with continuous treatment **eFigure 2. **Dynamic treatment effects with alternative outcomes **eTable 8. **Descriptive statistics of municipalities by school opening decision in the baseline period (as of end of September)

## References

[1] Auger KA, Shah SS, Richardson T, . Association between statewide school closure and COVID-19 incidence and mortality in the US. JAMA. 2020;324(9):859-870. doi:10.1001/jama.2020.14348 32745200 PMC7391181

[2] Donohue JM, Miller E. COVID-19 and school closures. JAMA. 2020;324(9):845-847. doi:10.1001/jama.2020.13092 32745182

[3] Azevedo JP, Hasan A, Goldemberg D, Iqbal SA, Geven K. Simulating the potential impacts of COVID-19 school closures on schooling and learning outcomes. Accessed March 25, 2021. https://openknowledge.worldbank.org/bitstream/handle/10986/33945/Simulating-the-Potential-Impacts-of-COVID-19-School-Closures-on-Schooling-and-Learning-Outcomes-A-Set-of-Global-Estimates.pdf

[4] Alsan M, Braghieri L, Eichmeyer S, Kim MJ, Stantcheva S, Yang D. Civil Liberties in Times of Crisis. National Bureau of Economic Research;2020. doi:10.3386/w27972

[5] Lichand G, Christen J. Using nudges to prevent student dropouts in the pandemic. Accessed March 24, 2021. https://papers.ssrn.com/sol3/papers.cfm?abstract_id=3724386

[6] Lichand G, Belchior C, Neto OL, Cossi J. The impacts of remote learning in secondary education: evidence from Brazil during the pandemic. Accessed July 21, 2021. https://papers.ssrn.com/sol3/papers.cfm?abstract_id=3841775

[7] Flasche S, Edmunds WJ. The role of schools and school-aged children in SARS-CoV-2 transmission. Lancet Infect Dis. 2021;21(3):298-299. doi:10.1016/S1473-3099(20)30927-0 33306982 PMC7837086

[8] Lessler J, Grabowski MK, Grantz KH, . Household COVID-19 risk and in-person schooling [published online March 1, 2021]. medRxiv. Posted March 1, 2021. doi:10.1101/2021.02.27.21252597 PMC8168618 33927057

[9] Lai S, Ruktanonchai NW, Zhou L, . Effect of non-pharmaceutical interventions to contain COVID-19 in China. Nature. 2020;585(7825):410-413. doi:10.1038/s41586-020-2293-x 32365354 PMC7116778

[10] Flaxman S, Mishra S, Gandy A, ; Imperial College COVID-19 Response Team. Estimating the effects of non-pharmaceutical interventions on COVID-19 in Europe. Nature. 2020;584(7820):257-261. doi:10.1038/s41586-020-2405-7 32512579

[11] Sun J, Shi Z, Xu H. Non-pharmaceutical interventions used for COVID-19 had a major impact on reducing influenza in China in 2020. J Travel Med. 2020;27(8):taaa064. doi:10.1093/jtm/taaa064 32324879 PMC7188119

[12] Seale H, Dyer CEF, Abdi I, . Improving the impact of non-pharmaceutical interventions during COVID-19: examining the factors that influence engagement and the impact on individuals. BMC Infect Dis. 2020;20(1):607. doi:10.1186/s12879-020-05340-9 32807087 PMC7430133

[13] Davies NG, Kucharski AJ, Eggo RM, Gimma A, Edmunds WJ; Centre for the Mathematical Modelling of Infectious Diseases COVID-19 working group. Effects of non-pharmaceutical interventions on COVID-19 cases, deaths, and demand for hospital services in the UK: a modelling study. Lancet Public Health. 2020;5(7):e375-e385. doi:10.1016/S2468-2667(20)30133-X 32502389 PMC7266572

[14] Regmi K, Lwin CM. Impact of non-pharmaceutical interventions for reducing transmission of COVID-19: a systematic review and meta-analysis protocol. BMJ Open. 2020;10(10):e041383. doi:10.1136/bmjopen-2020-041383 33093038 PMC7582337

[15] Cowling BJ, Ali ST, Ng TWY, . Impact assessment of non-pharmaceutical interventions against coronavirus disease 2019 and influenza in Hong Kong: an observational study. Lancet Public Health. 2020;5(5):e279-e288. doi:10.1016/S2468-2667(20)30090-6 32311320 PMC7164922

[16] Van Lancker W, Parolin Z. COVID-19, school closures, and child poverty: a social crisis in the making. Lancet Public Health. 2020;5(5):e243-e244. doi:10.1016/S2468-2667(20)30084-0 32275858 PMC7141480

[17] Fantini MP, Reno C, Biserni GB, Savoia E, Lanari M. COVID-19 and the re-opening of schools: a policy maker’s dilemma. Ital J Pediatr. 2020;46(1):79. doi:10.1186/s13052-020-00844-1 32517815 PMC7280677

[18] Viner RM, Russell SJ, Croker H, . School closure and management practices during coronavirus outbreaks including COVID-19: a rapid systematic review. Lancet Child Adolesc Health. 2020;4(5):397-404. doi:10.1016/S2352-4642(20)30095-X 32272089 PMC7270629

[19] Stein-Zamir C, Abramson N, Shoob H, . A large COVID-19 outbreak in a high school 10 days after schools’ reopening, Israel, May 2020. Euro Surveill. 2020;25(29). doi:10.2807/1560-7917.ES.2020.25.29.2001352 32720636 PMC7384285

[20] Kraemer MUG, Yang C-H, Gutierrez B, ; Open COVID-19 Data Working Group. The effect of human mobility and control measures on the COVID-19 epidemic in China. Science. 2020;368(6490):493-497. doi:10.1126/science.abb4218 32213647 PMC7146642

[21] Alon TM, Kim M, Lagakos D, VanVuren M. How should policy responses to the COVID-19 pandemic differ in the developing world? Accessed July 22, 2021. https://www.nber.org/papers/w27273

[22] Nugroho D, Pasquini C, Reuge N, Amaro D. COVID-19: How are countries preparing to mitigate the learning loss as schools reopen? trends and emerging good practices to support the most vulnerable children. Accessed July 21, 2021. https://www.unicef-irc.org/publications/pdf/COVID-19-How-are-Countries-Preparing-to-Mitigate-the-Learning-Loss-as-Schools-Reopen.pdf

[23] Gurdasani D, Alwan NA, Greenhalgh T, . Reopening schools without strict COVID-19 mitigation measures risks accelerating the pandemic. OSF Preprints. 2021. Posted March 6, 2021. doi:10.31219/osf.io/qg4bj

[24] Chang S, Pierson E, Koh PW, . Mobility network models of COVID-19 explain inequities and inform reopening. Nature. 2021;589(7840):82-87. doi:10.1038/s41586-020-2923-3 33171481

[25] Isphording IE, Lipfert M, Pestel N. School re-openings after summer breaks in Germany did not increase SARS-CoV-2 cases. Accessed March 19, 2021. https://COVID-19.iza.org/publications/dp13790/

[26] Amodio E, Battisti M, Kourtellos A, Maggio G, Maida CM. Schools opening and COVID-19 diffusion: evidence from geolocalized microdata. Accessed March 24, 2021. https://voxeu.org/article/school-openings-affect-local-covid-19-diffusion 10.1016/j.euroecorev.2021.104003 PMC8769565 35075308

[27] Bravata D, Cantor JH, Sood N, Whaley CM. Back to school: the effect of school visits during COVID-19 on COVID-19 transmission. Accessed July 27, 2021. https://www.nber.org/papers/w28645. 10.1016/j.jhealeco.2025.103025 PMC13616136 40554857

[28] Brasil.io. COVID-19: boletins informativos e casos do coronavírus por município por dia. Accessed March 24, 2021. https://brasil.io/dataset/covid19/caso_full/

[29] Governo do Estado de São Paulo. 3,5 milhões de alunos da rede estadual de SP finalizam o ano letivo de 2020 nesta quarta-feira. Accessed January 7, 2022. https://www.educacao.sp.gov.br/35-milhoes-de-alunos-da-rede-estadual-de-sp-finalizam-o-ano-letivo-de-2020-nesta-quarta-feira/

[30] Callaway B, Sant’Anna PHC. Difference-in-differences with multiple time periods. arXiv. Posted March 23, 2018. https://arxiv.org/abs/1803.09015.

[31] Bland JM, Altman DG. Regression towards the mean. BMJ. 1994;308(6942):1499. doi:10.1136/bmj.308.6942.1499 8019287 PMC2540330

[32] Ferrante L, Steinmetz WA, Almeida ACL, . Brazil’s policies condemn Amazonia to a second wave of COVID-19. Nat Med. 2020;26(9):1315. doi:10.1038/s41591-020-1026-x 32770168

[33] Lachassinne E, de Pontual L, Caseris M, ; COVIDOCRECHE collaborators. SARS-CoV-2 transmission among children and staff in daycare centres during a nationwide lockdown in France: a cross-sectional, multicentre, seroprevalence study. Lancet Child Adolesc Health. 2021;5(4):256-264. doi:10.1016/S2352-4642(21)00024-9 33571450 PMC9180428

[34] Waterfield T, Watson C, Moore R, . Seroprevalence of SARS-CoV-2 antibodies in children: a prospective multicentre cohort study. Arch Dis Child. 2021;106(7):680-686. doi:10.1101/2020.08.31.20183095 33172887

[35] Brookman S, Cook J, Zucherman M, Broughton S, Harman K, Gupta A. Effect of the new SARS-CoV-2 variant B.1.1.7 on children and young people. Lancet Child Adolesc Health. 2021;5(4):e9-e10. doi:10.1016/S2352-4642(21)00030-4 33581054 PMC7906637

[36] Engzell P, Frey A, Verhagen MD. Learning loss due to school closures during the COVID-19 pandemic. SocArXiv. Posted October 28, 2021. doi:10.31235/osf.io/ve4z7 PMC8092566 33827987

