# Supplementary material for: Association of COVID-19 Incidence and Mortality Rates With School Reopening in Brazil During the COVID-19 Pandemic
Source: JAMA Health Forum. 2022 Feb 11;3(2):e215032. doi: 10.1001/jamahealthforum.2021.5032 (PMC8903124; doi:10.1001/jamahealthforum.2021.5032)
Supplement: Supplement. — eTable 1. Summary of data sources eTable 2. Variable description eMethods. eAppendix. eFigure 1. Timeline of school reopening eTable 3. Heterogeneous treatment effects estimated through difference-in-differences eTable 4. Differences in cases for school-age children aggregated by cohort eTable 5. Differences in cases for parent-age cadults aggregated by cohort eTable 6. Difference-in-difference estimates aggregated by cohorts with matched sample eTable 7. Difference-in-difference model with continuous treatment eFigure 2. Dynamic treatment effects with alternative outcomes eTable 8. Descriptive statistics of municipalities by school opening decision in the baseline period (as of end of September) [file jamahealthforum-e215032-s001.pdf]

Supplemental Online Content

Lichand G, Doria CA, Cossi Fernandes JP, Leal-Neto O. Association of COVID-19 incidence and mortality rates with school reopening in Brazil during the COVID-19 pandemic. *JAMA Health Forum*. 2022;3(2):e215032. doi:10.1001/jamahealthforum.2021.5032

eTable 1. Summary of data sources

eTable 2. Variable description

eMethods.

eAppendix.

eFigure 1. Timeline of school reopening

eTable 3. Heterogeneous treatment effects estimated through differences-in-differences

eTable 4. Differences in cases for school-age children aggregated by cohort

eTable 5. Differences in cases for parent-age adults aggregated by cohort

eTable 6. Difference-in-differences estimates aggregated by cohorts with matched sample

eTable 7. Difference-in-differences model with continuous treatment

eFigure 2. Dynamic treatment effects with alternative outcomes

eTable 8. Descriptive statistics of municipalities by school reopening decision in the baseline period (as of end of September)

This supplemental material has been provided by the authors to give readers additional information about their work.

© 2022 Lichand G et al. *JAMA Health Forum.*

eTable 1: Summary of data sources

Name	Source	Year	Aggregation level
Population projections	IBGE	2019	municipality
Census data	IBGE	2010	municipality
School census	INEP	2019	municipality
Covid cases microdata	SUS	2020	municipality-week
Pandemic evolution	Brasil.io	2020	municipality-week
School status	SEDUC	2020	municipality-week

© 2022 Lichand G et al. *JAMA Health Forum.*

In eTable 2, we provide a description of all variables used in the paper.

eTable 2: Variable description

Variable	Description	Source
**Panel A:** dependent variables		
Log(cases)	Log of weekly new cases per thousand plus one	Pandemic evolution
Log(total cases)	Log of accumulated cases per thousand plus one	Pandemic evolution
Log(deaths)	Log of weekly new deaths per thousand plus one	Pandemic evolution
Log(total deaths)	Log of accumulated deaths per thousand plus one	Pandemic evolution
Cases for youngsters	Weekly cases for individuals with 13-18 years-old per thousand	Covid-19 cases microdata
Mobility	See separate description	Mobility reports
**Panel B:** other variables		
School reopening	Municipality was allowed to reopen schools	School status
Population	Municipality projected population in thousands (2019)	Population projection
Income	Average household per capita income	Census data
Number of schools	Total number of schools in the municipality	School census
Number of students	Total students per thousand in the municipality	School census
School infrastructure	See separate description	School census

© 2022 Lichand G et al. *JAMA Health Forum.*

eMethods.

Quality of school infrastructure

We combine different school characteristics to capture the quality of school infrastructure, using the principal component method.

We select the following variables from the 2019 Brazilian school census: the availability of bathrooms, school staff's bathrooms, shower, kitchen, garbage collection, piped water, basic sanitation, and the average number of students per class.

We select the first principal component as our composite index of school infrastructure, computed at the municipality level averaging across its schools. This first component accounts for approximately 40% of the total variance of the selected variables.

Mobility

Our mobility data is based off Google reports, computed from mobile-phone GPS information. Google calculates daily mobility information for more than 400 municipalities in the São Paulo state. As such, we have mobility data for roughly 78% of municipalities that reopened schools in 2020, and 56% of the ones that did not.

Google provides information concerning different types of mobility, including: 1) retail and recreation; 2) grocery and pharmacy, 3) parks, 4) transit stations; and 5) workplaces. We average across these five measures to generate an aggregate mobility index. We then average over that daily aggregate measure to generate a weekly municipal-level mobility index.

Mobility is expressed in percentage-point deviations from February 15, 2020, when Google started collecting mobility data.

Under-reporting of COVID-19 cases

As in several other developing countries, limited testing in Brazil has been linked to substantial under-reporting of COVID-19 cases. Estimates suggest that the actual number of COVID-19 cases could be three to ten times larger than that registered in official statistics[1].

Nevertheless, we do not expect under-notification to affect the findings of this study. The reason is two-fold. First, our estimates are based on a differences-in-differences strategy, which contrasts outcomes across municipalities that authorized schools to reopen and those that did not, before and after in-person activities could resume. As such, under-reporting would only bias our results if it were systematically different between groups and periods. Nonetheless, there is no reason why under-reporting should have been higher within municipalities that reopened schools, or for it to have increased after in-person activities could resume.

Second, and most importantly, this study provides first-hand evidence on the relationship between school reopening and COVID-19 deaths. Deaths tend to be much more precisely reported than cases; in effect, official statistics for COVID-19 related deaths tended to closely track the total number of excess deaths in 2020 and 2021, relative to previous years[2]. As we estimate a similar relationship for both cases and deaths, we conclude our results are not driven by under-reporting.

© 2022 Lichand G et al. *JAMA Health Forum.*

eAppendix.

Estimation method

To estimate the effect of school reopening on the outcomes of interest, we rely on the differences-in-differences approach. That is, we compare the trends of the dependent variables for treated and non-treated municipalities. However, recent literature suggests that a straightforward two-way fixed-effect regression is not appropriate in the context of an application with multiple periods and staggered treatment.

To circumvent this problem, we implement the Callaway and Sant'Anna procedure[3]. It can be seen as a three-step estimator. First, we divide the *treatment* group (the set of municipalities that authorized schools to reopen) into cohorts according to the week municipalities authorized schools to reopen. Let G_{gm}be a dummy variable indicating that municipality m reopened schools at period g and let C_m be a dummy variable indicating that municipality *m* did not authorize schools to reopen in 2020. Let N_g be the number of municipalities treated at time *g,* and *N* be the total number of time periods after treatment (=5 in our sample). Next, let $c = \{0,5,6,8,9\}$ be the set that identifies the weeks at which different cohorts were treated, in our sample. Then, in the first step of the procedure, we estimate:

$$G_{gm} = \Phi(X_m'\hat{\beta}), g \in c$$

where $\Phi(.)$ is the normal cumulative density function, X_m are municipal time-invariant characteristics. That is, we use a Probit model to predict how the likelihood of being treated at $g \in c$ relates to municipal characteristics (X_m'). Then, we calculate the propensity score of being treated in a certain period as the predicted probability from the first-stage ($\widehat{\boldsymbol{p_{gm}}}$).

Next, in the second step, we estimate cohort-time treatment effects, as follows:

$$\delta(g,t) = \sum_m \left(G_{gm} - w_{gm} * C_m\right) \frac{(Y_{mt} - Y_{mt-1})}{N_g}$$

where Y_{mt} is the outcome of interest in municipality m at time t, and w_{gm} is a weight computed as follows:

$$w_{gm} = \frac{\frac{\widehat{\boldsymbol{p_{gm}}}}{(\boldsymbol{1} - \widehat{\boldsymbol{p_{gm}}})}}{\sum_m \frac{\widehat{\boldsymbol{p_{gm}}}}{(\boldsymbol{1} - \widehat{\boldsymbol{p_{gm}}})}}$$

In words, the second step calculates difference-in-differences estimates by comparing units that were originally treated at time *g, t* periods later, to units in the control group -- attributing higher weight to observations that have larger probabilities of being treated based on their characteristics.

Lastly, once we estimate cohort-time treatment effects, we can aggregate them in multiple ways in the third step. In particular, it is possible to compute dynamics treatment effects as:

$$\delta_T = \sum_g \frac{\delta(g,T)}{N},$$

which is just the average of cohort-time treatment effects evaluated at *T*, which can even be negative (prior to the treatment onset , in the case of falsification tests). Alternatively, we could compute cohort-specific treatment effects as:

$$\delta_g = \sum_t \frac{\delta(g,t)}{N}$$

We compute standard errors using Callaway and Sant'Anna's standard procedure. This is done by block-bootstrapping standard errors at the municipality level. The block-bootstrap procedure allows arbitrary correlations between regression errors within the same municipality over time.

The fundamental identification assumption for the differences-in-differences strategy is that of *parallel trends* for the outcomes of interest. That is, we assume that, in the absence of school reopening, the log of each outcome would have followed the same time trend across both groups. We point out in the main text that there are significant initial differences between the two groups at the time of reopening. This does not invalidate the identification assumption, since differences-in-differences parses out any difference in outcomes across groups at the baseline period.

Some factors contribute to the plausibility of this assumption. First, the authorization to reopen schools is defined at the health region level, but the actual decision to reopen schools or not is made at the municipal level. As such, there was considerable variation in reopening decisions even within each health regions in the State. Moreover, the authorization to reopen schools was subject to the endorsement of local legislators, introducing randomness in the timing of the actual return of in-person school activities even conditional on municipal reopening decisions. We present falsification tests in Figure E3 whereby we test and fail to reject the presence of parallel trends *before school reopening*.

The Callaway and Sant'Anna estimator additionally requires that units that are *treated* cannot be *untreated*. For this reason, we drop from the analyses 2 municipalities that authorized schools to reopen, but reverse those decisions shortly after.

Nearest-neighbor matching

As a robustness check, we also implement a nearest-neighbor propensity score matching. We implement the procedure sequentially, starting the process by considering a sample consisting of the first cohort and the control group (never treated municipalities). We estimate a Probit model, as before:

$$G_{gm} = \Phi(X'_m\beta)$$

Then, we calculate the propensity score for this sample as the predicted value from the estimate above. We include the following variables as controls: per capita income, population, number of students, school infrastructure, and the number of baseline cases and deaths.

For each municipality in the *treated* cohort, we find the municipality in the *control* group with the closest propensity score, without replacement. After we match all municipalities in the first cohort, we build a new sample, including the second cohort of treatment and the control group without the municipalities matched in the first step of the process. Then, we re-estimate the Probit model for this alternative sample and match all municipalities in the second cohort. We repeat this procedure until we find a match to all cohorts of treatments.

We implement this sequential algorithm for two samples. First, the full sample of the study, including 643 São Paulo municipalities. Second, for the sub-sample of municipalities with available mobility data.

© 2022 Lichand G et al. *JAMA Health Forum.*

eFigure 1 starts by documenting the proportion of municipalities that reopened schools at different points in time, and the number of schools authorized to reopen as a result. The figure highlights that reopening decisions were staggered, with in-person school activities picking up only from November onwards; and even then, in-person school activities were far from universal in the State.

eFigure 1: Timeline of school reopening

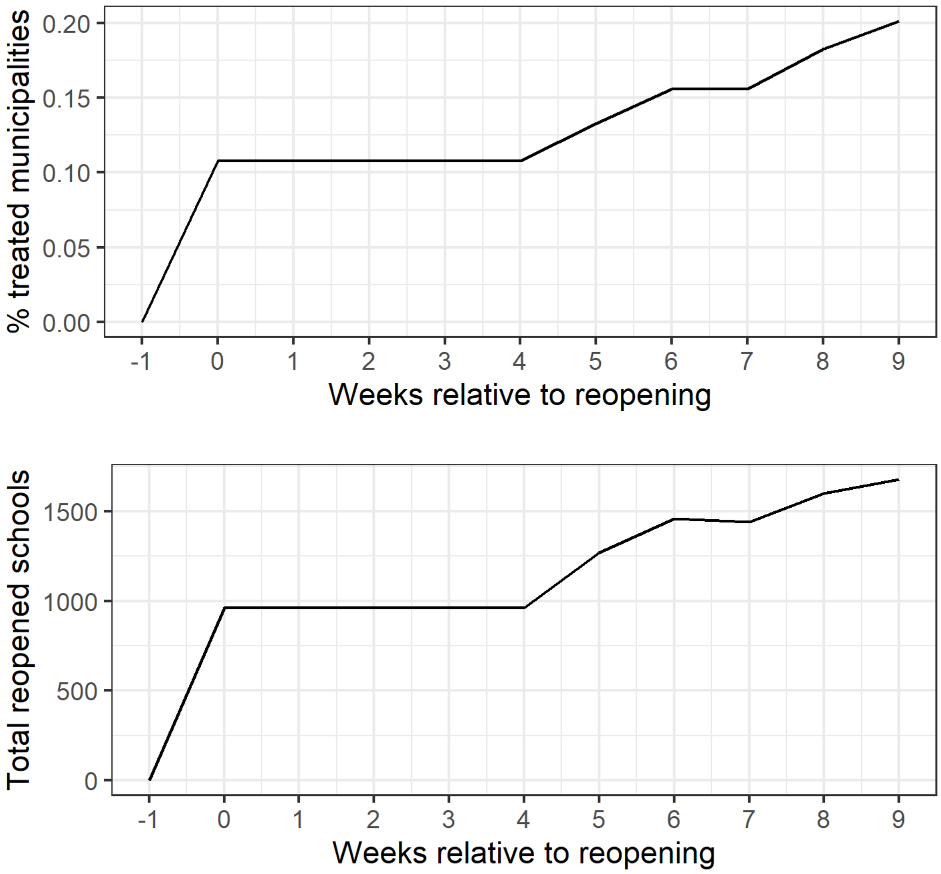

© 2022 Lichand G et al. *JAMA Health Forum.*

Next, eTable 3 estimates the relationship between school reopening and disease activity separately for below- and above-median (a) per capita income, (b) average quality of school infrastructure, (c) senior population share, and (d) baseline Covid-19 deaths. We find no statistically significant association even for the highest risk or most vulnerable sub-samples.

eTable 3 - Heterogeneous treatment effects estimated through differences-in-differences.

	log(weekly cases)	log(weekly deaths)
	(1)	(2)
**School infrastructure**		
Below median	0.037	0.005
	[-0.032, 0.105]	[-0.001, 0.010]
Above median	-0.009	-0.003
	[-0.080, 0.062]	[-0.014, 0.007]
**Per capita income**		
Below median	-0.051	0.002
	[-0.128, 0.027]	[-0.004, 0.007]
Above median	0.027	-0.001
	[-0.033, 0.070]	[-0.007, 0.005]
**65+ year-old share**		
Below median	-0.035	0.001
	[-0.095, 0.025]	[-0.002, 0.004]
Above median	0.031	0.003
	[-0.043, 0.104]	[-0.009, 0.016]
**Baseline severity of the pandemic**		
Below median	-0.066**	-0.001
	[-0.132, -0.024]	[-0.005, 0.003]
Above median	-0.023	-0.011
	[-0.123, 0.077]	[-0.026, 0.005]

© 2022 Lichand G et al. *JAMA Health Forum.*

eTable 4 estimates whether school reopening affected school-aged children using a triple-differences strategy, whereby we not only compare municipalities that authorized schools to reopen to those that did not, before and after in-person activities could resume, but also, school-aged children (ages 7-18) to young adults (ages 19-22) within each group and period. This strategy provides evidence on the direct effects of school reopening on COVID-19 incidence, since the latter age group should not be directly affected by school reopening. The analysis relies on additional data from COVID-19 testing (publicly available from SUS), which allows identifying patients' age. The limitation is that age-specific data are only available for cases, and only for specific weeks.

eTable 4: Differences in cases for school-age children aggregated by cohort

Cohort treatment	Cases for school-age children	
	Difference-in-differences	Tripple-difference
0	-0.007	0.002
	[-0.033, 0.020]	[-0.020, 0,024]
5	-0.004	0.007
	[-0.026, 0.019]	[-0.043, 0.057]
6	-0.005	-0.001
	[-0.022, 0.012]	[-0.022, 0.021]
Aggregate difference	-0.005	0.002
	[-0.018, 0.008]	[-0.012, 0.015]

© 2022 Lichand G et al. *JAMA Health Forum.*

eTable 5 estimates whether school reopening affected typical school parents using a similar triple-differences strategy, this time contrasting adults within the typical age range of primary and secondary school parents (ages 27-48) to older cohorts (ages 49-65).

eTable 5: Differences in cases for parent-age adults aggregated by cohort

Cohort treatment	Cases for parent-age adults	
	Difference-in-differences	Tripple-difference
0	-0.074	-0.020
	[-0.162, 0.013]	[-0.070, 0,031]
5	-0.037	-0.056
	[-0.146, 0.072]	[-0.169, 0.057]
6	0.068	0.098
	[-0.243, 0.379]	[-0.163, 0.359]

© 2022 Lichand G et al. *JAMA Health Forum.*

Next, eTable 6 re-estimates the differences-in-differences strategy for the matched sub-sample as a robustness check that results are not driven by different group characteristics.

e**Table 6:** Difference-in-differences estimates aggregated by cohorts with matched sample

Cohort treatment	log(weekly cases)	log(weekly deaths)	mobility
	(1)	(2)	(3)
0	0.011	0.000	1.254
	[-0.040, 0.063]	[-0.004, 0.003]	[-0.177, 2.864]
5	-0.177	-0.009	-1.280
	[-0.382, 0.028]	[-0.010, 0.029]	[-6.904, 4.343]
6	-0.052	0.002	0.022
	[-0.214, 0.110]	[-0.007, 0.011]	[-1.666, 1.710]
8	-0.175	-0.007	4.180***
	[-0.371, 0.021]	[-0.027, 0.014]	[1.010, 7.365]
9	0.025	-0.006	-0.062
	[-0.206, 0.257]	[-0.020, 0.008]	[-4.774, 4.651]
Aggregate difference	-0.043	0.000	0.966
	[-0.098, 0.013]	[-0.005, 0.004]	[-0.283, 2.215]

© 2022 Lichand G et al. *JAMA Health Forum.*

Next, eTable 7 estimates the relationship between school reopening and COVID-19 cases and deaths more flexibly, allowing for the possibility that the decision to reopen schools locally might also affect disease activity in neighboring municipalities. To do that, we implement a fixed-effects estimator with a continuous treatment variable, defined as the negative of the log distance to nearest municipality that reopened schools at each week (=0 if the municipality itself has authorized schools to reopen then). This specification also does not detect a systematic relationship between proximity to schools authorized to reopen and local disease activity.

eTable 7: Difference-in-differences model with continuous treatment

	log(weekly cases)	log(weekly deaths)
log(proximity to closest treated	-0.034***	-0.001
municipality)	[-0.044, -0.024]	[-0.003, 0.001]

© 2022 Lichand G et al. *JAMA Health Forum.*

Last, eFigure 2 presents robustness checks for the log transformation performed in the outcome variables. In the main analysis, we show results for the log of COVID-19 cases and deaths adding 1 to all observations, to prevent municipality-week pairs with zero cases or deaths to be dropped from the analyses. Here, we consider two alternative specifications. First, we estimate dynamic treatment effects for the log of new cases and deaths dropping observations for which new cases or death equal zero. Second, we estimate dynamic treatment effects for the outcome variables in levels, that is, without taking the log transformation, and using all observations. Our conclusions are robust to these alternative specifications.

e**Figure 2:** Dynamic treatment effects with alternative outcomes

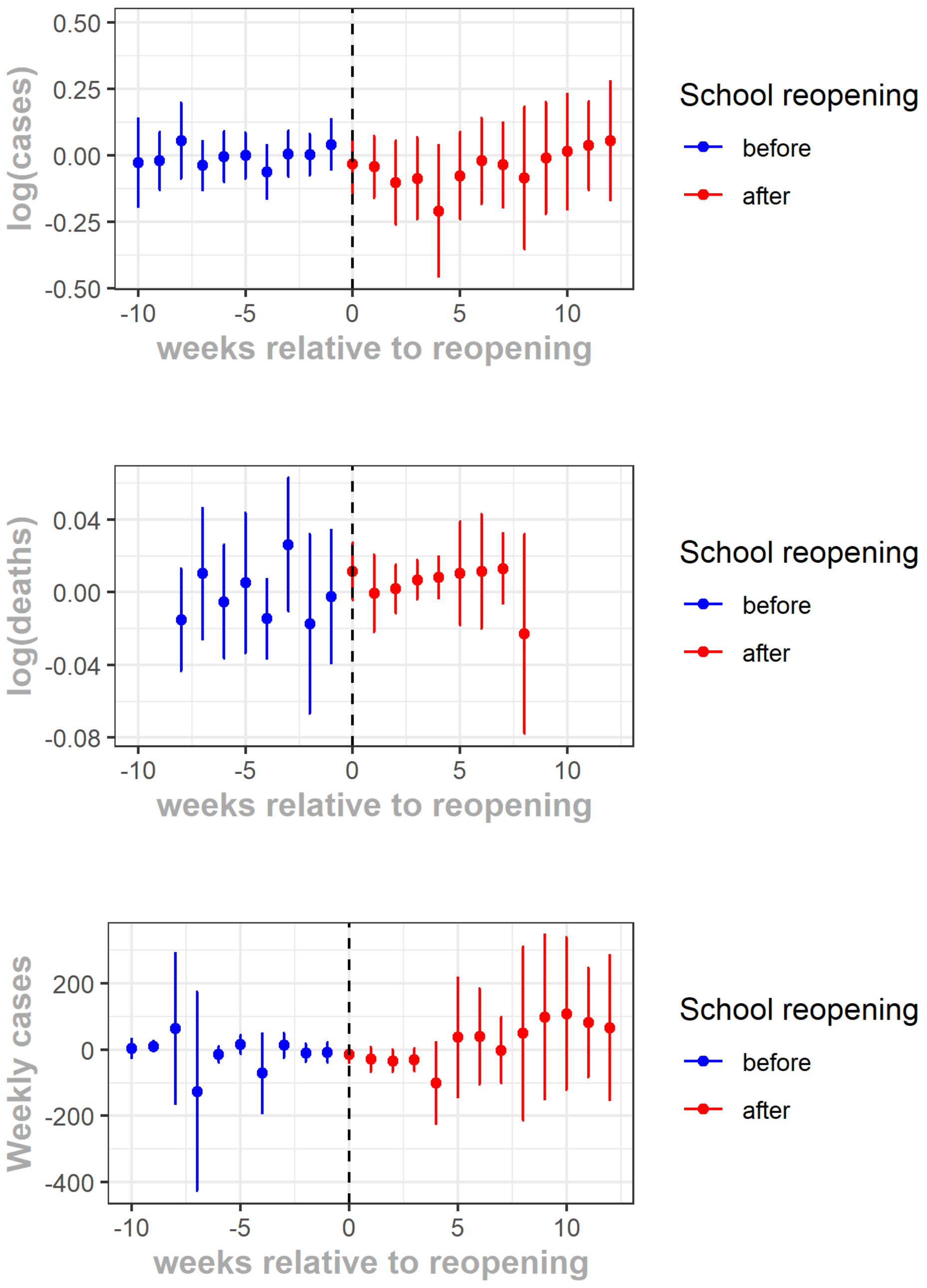

© 2022 Lichand G et al. *JAMA Health Forum.*

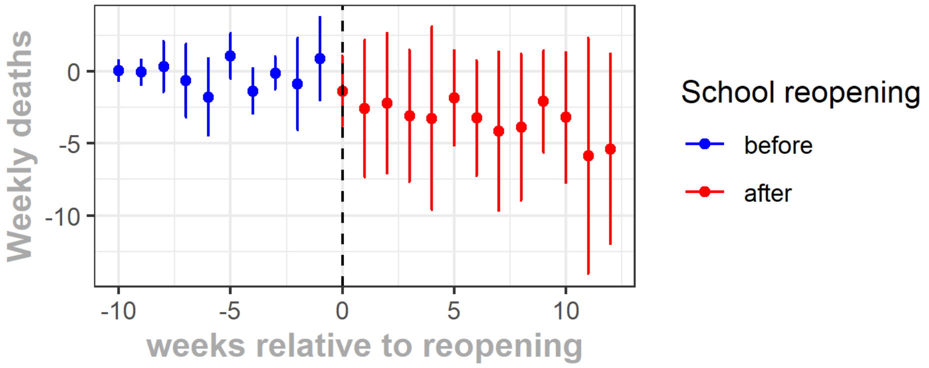

© 2022 Lichand G et al. *JAMA Health Forum.*

eTable 8. Descriptive Statistics of Municipalities by School Opening Decision in the Baseline Period (as of end of September)

	**Did not reopen schools**	**Reopened schools**
New		
Cases per thousand	0.79	0.76
Deaths per thousand	0.03	0.02
Accumulated deaths per thousand	0.44	0.49
Income per capita	672.17	804.58
Population		
Thousands	38.65	200.37
Density, hab/km^2	277.77	586.37
No. of		
Schools	19.41	67.94
Students, thousands	7.21	34.34
School infrastructure	–0.01	0.00
Municipalities	514	129

This table presents averages for several variables, separately for municipalities that reopened schools at any point of 2020 and those that did not. Time-variant variables are measured at the baseline period (last week of September 2020). Data for Covid-19 cases and deaths (rows 1-3) come from Brasil.io. Municipal characteristics come from the Brazilian 2010 census (rows 4-6). School characteristics comes from the Brazilian 2019 school census (rows 7-9).

© 2022 Lichand G et al. *JAMA Health Forum.*

eReferences

1. Prado, MF, BBP Antunes, LSL Bastos, IT Peres, AAB da Silva, LF Dantas, FA Baião, P Maçaira, S Hamacher, FA Bozza. Análise da subnotificação de COVID-19 no Brasil. Rev. bras. ter. intensiva , 32 (2) 2020.

2. Fujiwara, T. Estimating Excess Deaths due to Covid-19 in Brazil using the "Cartorios" Data. Available at: http://www.princeton.edu/~fujiwara/papers/excess_deaths.pdf.

3.Callaway B, Sant'Anna PHC. Difference-in-differences with multiple time periods [Internet]. arXiv [econ.EM]. 2018. Available from: http://arxiv.org/abs/1803.09015.

4. Callaway B, Sant'Anna PH. Difference-in-differences with multiple time periods. Journal of Econometrics. 2021 Dec 1;225(2):200-30.

© 2022 Lichand G et al. *JAMA Health Forum.*
